# Dietary patterns of adults and their associations with Sami ethnicity, sociodemographic factors, and lifestyle factors in a rural multiethnic population of northern Norway - the SAMINOR 2 clinical survey

**DOI:** 10.1186/s12889-019-7776-z

**Published:** 2019-12-04

**Authors:** Natalia Petrenya, Charlotta Rylander, Magritt Brustad

**Affiliations:** 1 Department of Community Medicine, Faculty of Health Sciences, University of Tromsø The Arctic University of Norway, Postboks 6050 Langnes, 9037 Tromsø, Norway; 2 The Public Dental Health Service Competence centre of Northern Norway, TkNN, Tromsø, Norway

**Keywords:** Dietary patterns, Sami indigenous people, Principal component analysis, Sociodemographic characteristics, Lifestyle factors, Northern Norway

## Abstract

**Background:**

Few population-based studies have assessed dietary behaviors in the rural multiethnic population of Northern Norway. The present study determined dietary patterns and investigated their association with Sami ethnicity, sociodemographic factors, and lifestyle factors in a multiethnic population in rural Northern Norway.

**Methods:**

This cross-sectional study included 4504 participants of the SAMINOR 2 Clinical Survey (2012–2014) aged 40–69 years. All participants completed a lifestyle and food frequency questionnaire. Dietary patterns were determined using principal component analysis. Associations between food patterns and ethnicity, sociodemographic factors, and lifestyle factors were examined by multiple linear regression.

**Results:**

Six dietary patterns were identified that accounted for 28% of the variability in food intake in the study sample: ‘processed meat/westernized’, ‘fish/traditional’, ‘fruit/vegetables’, ‘reindeer/traditional’, ‘bread and sandwich spreads’, and ‘sweets and bakery goods’. The ‘reindeer/traditional’ pattern was most common among the inland Sami population. The ‘fish/traditional’ pattern was most common among costal multiethnic Sami and least common among inland Sami and among women independent of ethnicity. The ‘fish/traditional’ pattern was also positively associated with older age, high education level, small household size, and smoking. Adherence to the ‘processed meat/westernized’ pattern was lower among inland Sami than inland/coastal non-Sami; no ethnic differences in adherence to this pattern were found between costal multiethnic Sami and inland/coastal non-Sami. Unhealthy lifestyle factors, like low physical activity level and smoking, and younger age were mainly associated with the ‘processed meat/westernized’ pattern, whereas socioeconomic factors like low education, low gross annual household income, and large household size were related to the ‘sweets and bakery goods’ pattern. Male gender, low education level, and smoking were associated with the ‘bread and sandwich spreads’ pattern. The ‘fruit/vegetables’ pattern was characterized by healthy dietary choices and a health-conscious lifestyle, and was more common in women with a high education level and income.

**Conclusions:**

Adherence to the six identified dietary patterns was characterized by different sociodemographic and lifestyle factors. Ethnicity, in combination with geographical region of residence, was associated with dietary behaviors. This study provides knowledge that will be useful in future studies on dietary patterns related to chronic diseases in the rural population of Northern Norway.

## Background

**Fig. 1:**
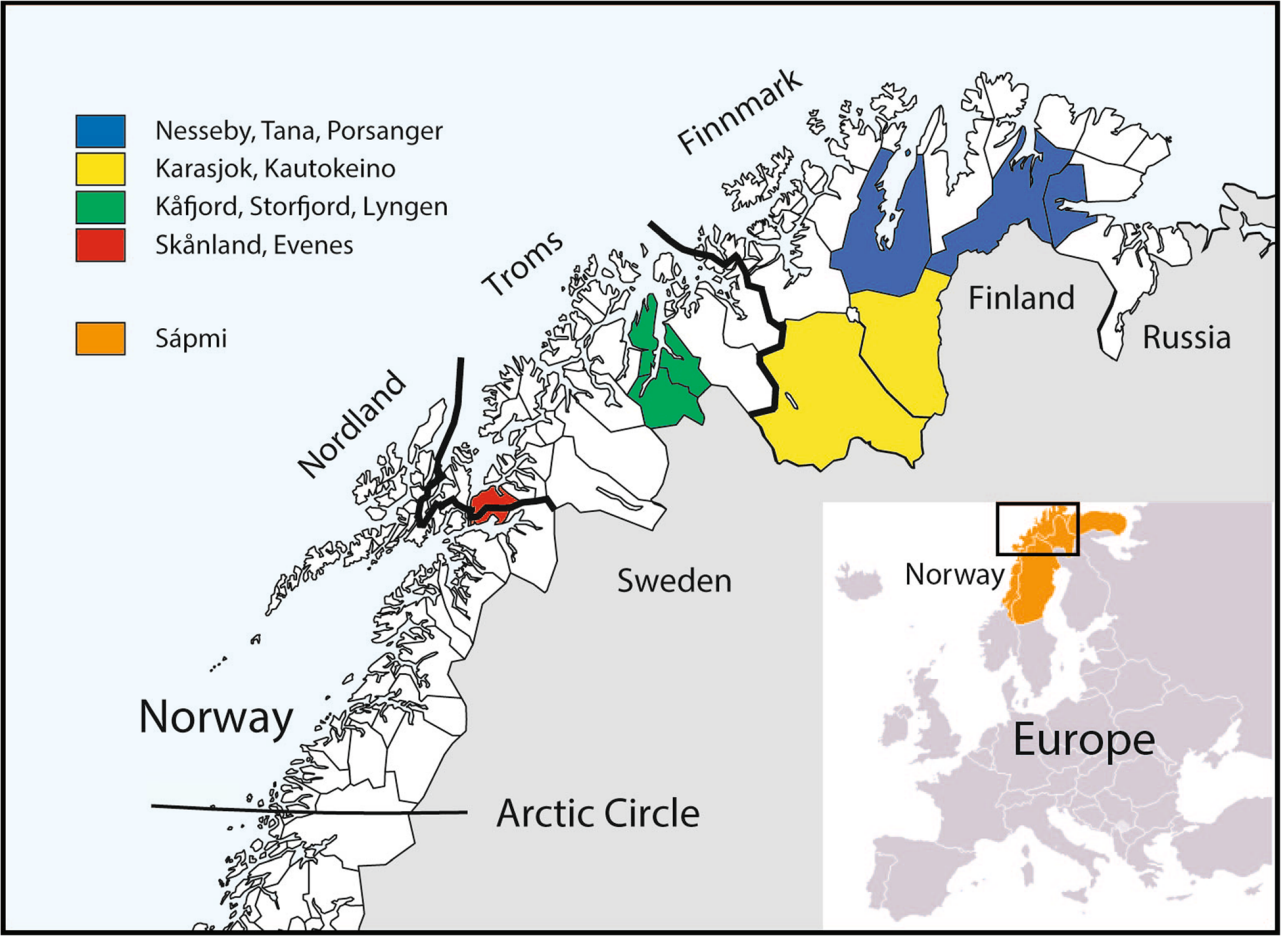
A map of Norway north of the Arctic Circle. The three northernmost counties in Norway (Finnmark, Troms, and Nordland) are indicated in the map as well as the 10 selected municipalities included in the SAMINOR 2 Clinical Survey, 2012–2014. Footnotes: The 10 selected municipalities are presented using different colors according to their geographic location and listed in the upper left corner: 1) Yellow – Karasjok and Kautokeino municipalities represent the inland part of Finnmark County; 2) Blue – Tana, Nesseby, and Porsanger municipalities represent the coastline of Finnmark County; 3) Green – Kåfjord, Storfjord, and Lyngen municipalities represent the coastline of the northern part of Troms County; 4) Red – Skånland and Evenes municipalities represent the coastline of the southern part of Troms County and Nordland County. Sápmi, the traditional Sami settlement area (Norway, Sweden, Finland, Russia’s Kola Peninsula), is presented in orange color. The map of the study area is used with permission from the Centre for Sami Health Research (CSHR) at UiT The Arctic University of Norway. It was designed by Marita Melhus at CSHR, based on a raw map of Norway from the Norwegian Mapping Authority, and a map of Europe and Sápmi which has been released to the public domain at Wikipedia. The first time a version of this map was used was in the paper by Kvaloy et al. [1]

The Sami are the only ethnic group in Norway that is acknowledged as Indigenous People by the Norwegian State. The Sami are an ethnic minority group that live in Sweden, Finland, Russia (Kola Peninsula), and Norway, which has the largest Sami population. However, the Sami are a majority population in some of the municipalities, like Karasjok and Kautokeino, in the innermost parts of Finnmark County in Northern Norway, where reindeer herding is common (Fig. 1). Historically, the livelihood of the Sami was based on reindeer herding, fishing, and small-scale agriculture, depending on the geographical region of residence. The diet of Sami people was mainly based on foods from the local environment and contained large amounts of reindeer and fish [2, 3].

The Sami people have lived alongside and had interactions with Norwegians and the populations from neighboring countries for thousands of years, while preserving their unique culture. Throughout the 19th and 20th centuries, the Sami were exposed to a long and extensive assimilation process. This has caused a weakening of Indigenous culture and identity of the Sami due to the strong influence of the country in which they resided, through initiatives like schooling exclusively in the national language and prohibitions against using the Sami language at school. Coastal Sami were the most strongly affected, and historically many chose to hide their ethnic affiliation. Following the recognition and protection of the rights of ‘being Sami’ in the last decades by the Norwegian government, the situation has improved. Indeed, compared with vast inequality between Indigenous and non-Indigenous people globally [4], no, or only minor differences in socioeconomic status (SES), nutrition, health, and life expectancy have been consistently reported between the Sami and their non-Indigenous counterparts in Norway [5–7].

Reindeer herding and a traditional Sami lifestyle remain important in Sami culture and food traditions, but today the Sami are a heterogeneous population in terms of occupation and lifestyle. Assimilation, as well as social, economic, environmental, and cultural changes have resulted in nutrition transitions, including a declining consumption of nutrient-dense traditional Sami foods [8], which are an excellent source of many essential nutrients like iron, vitamin B12, zinc, selenium, polyunsaturated fatty acids, and vitamin D [9, 10]. It was recently shown that Sami ethnicity and geographical region of residence are related to the consumption of culturally-specific individual food items and food groups [8]. However, little is known about the association between overall diet – as estimated using dietary patterns – and Sami ethnicity, sociodemographic factors, and lifestyle factors, in the population of rural Northern Norway, which has a mixed Sami and Norwegian population.

Dietary pattern analyses has become widely used in nutritional epidemiology [11]; it uses a priori and a posteriori approaches [12, 13]. A posteriori dietary patterns are derived by statistical methods, such as factor or cluster analysis, and may provide a better description of the actual diet of a specific population group. Principal component analysis (PCA), a form of factor analysis, derives linear combinations of foods based on their intercorrelations [14]. This method is suitable for large population-based studies using food frequency questionnaire (FFQ) data, shows good reproducibility and validity [15–19], and may have advantages over cluster analysis with respect to the interpretability of the resulting dietary patterns [17]. However, arbitrary decisions, such as the use of predefined food groups, sample-specific results, and limited comparability, are some of the methodological challenges of this approach.

Dietary patterns have previously been studied in the Population-based Study on Health and Living Conditions in Regions with Sami and Norwegian Populations (SAMINOR 1, 2003–2004) applying PCA and a two-step clustering method and using self-reported frequency of consumption of a limited number of selected foods [2, 3, 20]. This approach identified five dietary patterns: ‘reindeer’, ‘fish’, ‘average’, ‘fruits and vegetables’, and ‘westernized, traditional marine’. The ‘reindeer’ pattern is a very specific to these geographic regions and was highly prevalent among individuals with strong Sami affiliation, those residing in the inland region, and those with obesity, i.e., a body mass index (BMI) ≥30 kg/m^2^ [3]. The ‘reindeer’ pattern was also associated with more favorable iron stores in men and women [20]. The other four patterns identified were more common in participants with Norwegian ethnicity and in those residing in the coastal region. The ‘fruit and vegetables’ pattern was dominated by women and was characterized by a health-conscious lifestyle [3].

The rural Sami and non-Sami population of Northern Norway have a high prevalence of obesity and diabetes [21–24], both of which are related to diet and are associated with increased health risks. The second wave of the Population-based Study on Health and Living Conditions in Regions with Sami and Norwegian populations (SAMINOR 2) was conducted to follow up on the prevalence of and factors associated with chronic diseases in rural Northern Norway, and included the use of a comprehensive semi-quantitative FFQ. It has been suggested that rapid changes in the diet of certain Indigenous populations may partly explain the dramatic increase in prevalence of diet-related chronic diseases [25–27]. Taking into account the high prevalence of central obesity and diet-related chronic conditions like diabetes in the Sami population, studies on dietary patterns and related factors are useful. Therefore, the present study determined dietary patterns and investigated their associations with Sami ethnicity, sociodemographic factors, and lifestyle factors in a multiethnic population residing in rural Northern Norway.

## Methods

### Study design and population

The present analysis is based on the SAMINOR 2 Clinical Survey, a cross-sectional study performed by the Centre for Sami Health Research, UiT The Arctic University of Norway in 2012–2014, with data collection from 10 municipalities (Fig. 1). The methods of SAMINOR 2 Clinical Survey have been described in detail elsewhere [28]. In brief, all inhabitants aged 40–79 years and residing in these municipalities were invited to participate in the study by post; the mailing included a personal invitation, an information letter, an eight-page questionnaire, and an appointment for a clinical exam. Participants were asked to complete the questionnaire, which contained a four-page FFQ, and bring it to the clinical exam, which consisted of a short clinical examination and blood collection.

As per the methods of a previous analysis carried out by our group [7, 8, 29], our sample considers only the 10,399 invitees aged 40–69 years, of whom 4876 participated in the study (participation rate 47%). Participants were excluded if: (1) they did not report ethnicity (*n* = 115); (2) they only reported non-Western European, Asian, and African ethnic origins (*n* = 69), because it was assumed that the FFQ was not valid for these participants due to different food cultures; (3) the FFQs was incomplete (*n* = 91) i.e., half of the questions in the FFQ were left blank; (4) height and weight measurements were missing (*n* = 7). Finally, we excluded participants who were within the top and bottom 1% of energy intake/basal metabolic rate ratios (*n* = 90) to account for overreporting or underreporting [30, 31]. Thus, the final study sample consisted of 4504 participants.

### Dietary assessment

We have provided the description of dietary assessment method elsewhere [7, 8]. Briefly, minor adjustments, mainly related to some known traditional food items, were implemented in the Norwegian Women and Cancer (NOWAC) study FFQ; this slightly modified version of FFQ was applied in the SAMINOR 2 Clinical Survey. Adjustments consisted of including some traditional food items (fresh water fish, halibut, moose meat, grouse and other game birds, seagull eggs, food made with animal blood, i.e., black pudding from lamb/sheep, cattle, reindeer, or moose), as well as the modification of questions regarding reindeer meat, eggs, potato, and water consumption (www.saminor.no). The frequency of consumption of the foods and beverages included in the FFQ were reported for the past 12 months. We used the NOWAC study nutrient calculation program to estimate daily intake of foods in grams per day (g/day). The NOWAC FFQ has previously been validated for the general female population of Norway and is described in detail elsewhere [32–34].

### Classification of ethnicity, geographic region of residence, sociodemographic factors, and lifestyle factors

Age was divided into three groups: 40–49 (reference group), 50–59, and 60–69 years. Ethnicity was classified as non-Sami, including participants who considered themselves as Norwegian, Kven, or immigrants from Western European countries; multiethnic Sami, including participants who defined themselves as Sami in combination with any other ethnic background; or Sami, which included participants who defined themselves as Sami only. Geographic region of residence was categorized as the inland region (including the municipalities of Karasjok and Kautokeino) and the coastal region (including the other eight municipalities), based on whether or not the municipalities include coastal areas (Fig. 1). Participants were then divided by geographic region into the following five ethnic/geographic groups: inland Sami, inland multiethnic Sami, coastal Sami, coastal multiethnic Sami, and non-Sami (including inland and coastal; reference group). Education level was divided into four groups according to number of years of education: 0–9 years (reference group), 10–12 years, 13–16 years, and ≥ 17 years. Three gross annual household income groups were used in the analysis: low (≤450,000 NOK; reference group), average (451,000–750,000 NOK), and high (> 750,000 NOK). Household size was categorized as: 1 person (reference group), 2 persons, 3–4 persons, and 5–8 persons. Participants reported their physical activity level on a scale from 1 to 10, where 1 corresponded to a “very low” and 10 corresponded to a “very high” physical activity level. The question on physical activity has been validated against objective measures in another questionnaire-based study of Norwegian women [35]. Physical activity level was categorized as low (1–3; reference group), moderate (4–7), and high (8–10). Smoking status was categorized as current, former, and never (reference group).

### Height, weight, and body mass index

Height and weight were measured during the clinical exam using an electronic Height, Weight & Fatness Measuring System device (DS-103, Dongsahn Jenix, Seoul, South Korea), with the participants wearing light clothing and no shoes. Height was measured to the nearest 0.1 cm, and weight to the nearest 100 g. BMI was then calculated in kg/m^2^. BMI was classified into three groups: underweight and normal weight (BMI < 24.9 kg/m^2^; reference group), overweight (BMI 25–29.9 kg/m^2^), and obesity (BMI ≥ 30.0 kg/m^2^).

### Statistical methods

**Fig. 2:**
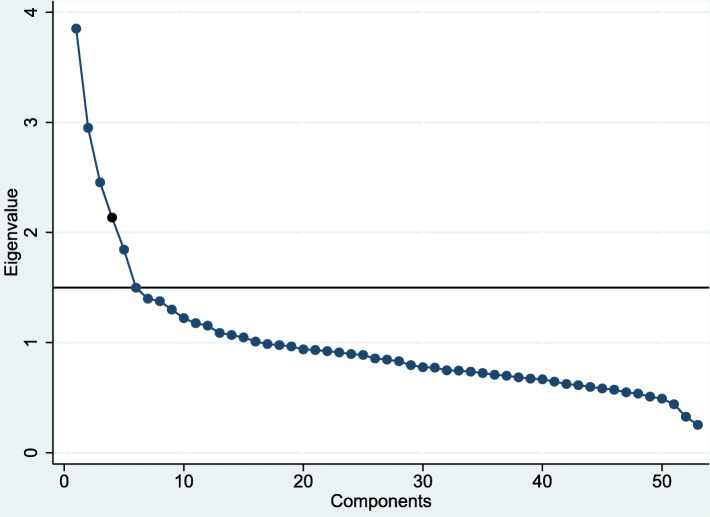
Scree plot for identification of dietary patterns (components) by principal component analysis. Food intakes (g/day) were aggregated into 53 food groups and used as input variables. Factors considered appropriate for the patterns shown in Table 2 are the six factors with eigenvalues > 1.5

We merged food items from the FFQ into 53 predefined food groups, taking into consideration similarities in nutrients and ingredients, as well as their use in the diet. Several foods (for example, yogurt, reindeer meat, and eggs) were not merged because it was inappropriate. Dietary patterns were analyzed by PCA based on the 53 merged food groups (Additional file 1 Table S1). The Kaiser-Meyer-Oklin measure of sampling adequacy (0.714) and the Bartlett test of sphericity (*p* < 0.001) confirmed the appropriateness of the data. We chose the number of components best describing the data based on the scree plot (a break of the slope) (Fig. 2), the interpretability of the factor loadings, and higher than 1.5 eigenvalues. Varimax orthogonal rotation was performed by generating nonrelated factors in order to achieve better interpretability of dietary patterns. Rotated factor loadings with absolute values of > 0.15 were considered to contribute to a pattern, and therefore reported. Food groups that loaded highly on the principal component were considered when identifying a name for each of the six dietary pattern components. Scores for these six retained components were calculated for each participant. We performed a sensitivity analysis by conducting the PCA in two random halves of the dataset. This analysis yielded the same dietary patterns, and only small differences were observed with respect to factor loadings (data not shown). Another sensitivity analysis was performed by gender, and showed that the food groups that significantly contributed to the dietary patterns were similar, and their factor loadings were comparable between men and women. Therefore, the entire sample was used as the analytical sample.

To determine the sociodemographic and lifestyle factors associated with the different dietary patterns, we used multiple linear regression. Age, gender, ethnic/geographic group, education level, gross annual household income, household size, physical activity level, smoking status, BMI, and energy intake were used as covariates in the regression models.

The assumptions of the linear regression models were met. We used list-wise deletion to handle missing data. We present adjusted parameters estimates and 95% confidence intervals for each model. We also tested linear trends of the dietary component scores across the ordered levels of age, education level, gross annual household income, household size, physical activity level, and BMI using trend contrasts. *P*-values < 0.05 were considered statistically significant, and all statistical tests were two-sided. Data were analyzed using STATA version 14 (StataCorp, College Station, TX, USA).

## Results

### Characteristics of the study sample

The mean age of the participants was 55.9 (standard deviation 18.5) years. There were slightly more women (54.4%) than men, and more participants aged 60–69 years (40.5%) than aged 40–49 and 50–59 years. The study sample consisted of 60.9% non-Sami, 16.8% inland Sami, 2.6% inland multiethnic Sami, 8.5% coastal Sami, and 11.2% coastal multiethnic Sami (Table 1).

**Table 1:** Characteristics of the study participants (*n* = 4504)^a^. The SAMINOR 2 Clinical Survey, 2012–2014

Characteristics	Number	Percent
Age (years)		
40–49	1212	26.9
50–59	1467	32.6
60–69	1825	40.5
Gender		
Men	2054	45.6
Women	2450	54.4
Ethnic/geographic group		
Inland and coastal non-Sami	2743	60.9
Inland Sami	755	16.8
Inland multiethnic Sami	119	2.6
Coastal Sami	384	8.5
Coastal multiethnic Sami	503	11.2
Education level (years)		
0–9	919	21.1
10–12	1372	31.5
13–16	1245	28.6
≥ 17	817	18.8
Gross annual household income (NOK)		
Low (≤450,000)	1278	30.6
Average (451,000–750,000)	1508	36.1
High (> 750,000)	1397	33.4
Household size		
1 person	719	16.3
2 persons	1986	45.0
3–4 persons	1274	28.9
5–8 persons	432	9.8
Physical activity level		
Low	850	19.4
Medium	2801	63.9
High	734	16.7
Smoking status		
Never	1608	36.0
Former	1935	43.4
Current	920	20.6
BMI category		
Underweight/normal weight	1192	26.5
Overweight	2018	44.8
Obesity	1294	28.7

^a^Subgroups may not total 4504 due to missing values. Missing values: *n* = 151 for education level, *n* = 321 for gross annual household income, *n* = 93 for household size, *n* = 41 for smoking status, *n* = 119 for physical activity level

### Principal component analysis

We identified six dietary patterns, which explained 27.9% of the variability in food intake in the study sample. The results of the PCA are presented in Table 2. The first dietary pattern was named ‘processed meat/westernized’ because of positive loadings for processed meat/meat dishes, pizza with meat toppings, pasta and rice, chicken, red meat (beef, pork, and mutton), sauces (for fish, meat/pasta dishes), salty snacks, squash/lemonade/soft drinks, eggs, and white bread. The second dietary pattern was named ‘fish/traditional’ due to high positive loadings for lean fish and traditional fish roe/liver. In addition, this pattern was characterized by positive loadings for oily fish, sauces, fat and sour cream eaten together with fish, shellfish, potatoes, seagull eggs or eggs of other seabirds, and three types of alcoholic beverages (i.e., spirits, wine, and beer). The third dietary pattern was called ‘fruit/vegetables’ because of the high positive loadings for vegetables and fruit/berries. In addition, this pattern was characterized by positive loadings for water, oily fish, breakfast cereals, crispbread, porridges other than rice (oatmeal, etc.), white cheese, and yogurt. Negative loadings were detected for spirits and beer. The fourth dietary pattern was named ‘reindeer/traditional’ based on the following traditional local foods: reindeer meat, food made with animal blood, freshwater fish, unfiltered/boiled coffee, game meats, and soup. In addition, this pattern was characterized by higher consumption of milk/cream and sugar added to coffee or tea. The fifth dietary patterns was named ‘bread and sandwich spreads’ because this pattern loaded high for coarse/semi coarse bread, bacon, preserved meats (salami, ham, etc.), liver pâté, white cheese, and fat on bread. In addition, this pattern was characterized by positive loadings for potatoes, low-fat/skimmed milk (regular or sour/fermented/cultured), mayonnaise-based salads, and whey cheese, and negative loadings for breakfast cereals. The sixth dietary pattern was named ‘sweets and bakery goods’ and was characterized by high intake of bakery goods (i.e., yeasted bakery goods (buns, etc.), Danish pastries, cakes, pancakes, waffles, cookies, biscuits, a traditional Norwegian soft bread/mashed potato flatbread (‘lefser/lomper’ in Norwegian), and sugar-rich food items (i.e., sweets/candy, chocolate, chocolate/caramel pudding, rice pudding/creamed rice, mousse/fromage, compote, stewed fruit, canned fruit, jam sandwich spread). There were also positive loadings for rice porridge, whey cheese, breakfast cereals, yogurt, and potatoes, and negative loadings for shellfish, bacon, preserved meats (salami, ham, etc.), liver pâté, eggs, beer, and wine.

**Table 2:** Factor loadings for food items/defined food groups that loaded >|0.15| in varimax rotated principal components

Food groups	Dietary patterns					
	Processed meat/ westernized	Fish/traditional	Fruit/vegetables	Reindeer/traditional	Bread and sandwich spreads	Sweets and bakery goods
Whole milk (regular or sour/fermented/cultured)						
Low-fat/skimmed milk (regular or sour/fermented/cultured)					0.22	
White cheese, full and low fat			0.19		0.29	
Whey cheese, full and low fat					0.17	0.26
Milk or cream to coffee or tea				0.22		
Full fat and low fat sour cream for fish meals/dishes		0.20				
Yogurt			0.21			0.17
Breakfast cereals			0.29		−0.17	0.19
Rice porridge						0.38
Other porridge; oatmeal, etc.			0.25			
Coarse/semi coarse bread					0.51	
Crispbread			0.27			
White bread	0.16					
Fat as spread on bread (butter, margarine and their blends)					0.20	
Fat as spread on bread (light/olive oil margarine)					0.29	
Bacon, preserved meats (salami, ham, etc.), liver pâté (topping on bread)					0.40	−0.17
Mayonnaise-based salads (sandwich spreads/fillings)					0.24	
Vegetables			0.37			
Potatoes		0.20			0.21	0.17
Fruit and berries			0.36			
Lean fish (filets/steaks)		0.40				
Oily fish, filets/steaks and fish/spreads on bread (smoked/canned)		0.25	0.23			
Freshwater fish				0.30		
Fish products		0.24				
Shellfish (i.e., prawns/shrimp, crabs, mollusks)		0.21				−0.19
Fish roe/liver		0.37				
Liquid (bottled) cod liver oil						
Seagull eggs or eggs of other seabirds		0.19				
Red meat (beef, pork, and mutton)	0.25					
Reindeer meat				0.48		
Game meats (moose meat, grouse, and other game birds)				0.23		
Food made with animal blood				0.37		
Pizza with meat toppings	0.39					
Processed meat/meat dishes	0.40					
Sauces for fish, meat/pasta dishes	0.24	0.17				
Melted/solid butter/margarine for fish meals/dishes		0.25				
Chicken	0.31		0.16			
Pasta and rice	0.32					
Soup				0.23		
Eggs	0.18					−0.18
Bakery goods						0.38
Sweets, ice cream, chocolate, desserts						0.35
Sugar to coffee and tea				0.21		
Salty snacks	0.29					
Water (tap/bottled)			0.34			
Coffee, except boiled				−0.18		
Unfiltered/boiled coffee				0.38		
Juice						
Squash/lemonade/soft drink containing sugar and without sugar	0.21					
Beer/alcopops		0.19	−0.16			−0.16
Wine		0.17				−0.19
Liqueur/fortified wine						
Liquor/distilled spirits		0.25	−0.18			
Eigenvalue	3.9	3.0	2.5	2.2	1.8	1.5
% of variance explained by each component	7.3	5.6	4.6	4.1	3.5	2.8
% of variance explained by 6 components	27.9					

### Linear regression analysis

Table 3 summarizes the results from six multivariable regression models exploring the relationships between dietary component scores and sociodemographic/lifestyle characteristics.

**Table 3:** Multiple linear regression^a^ exploring associations between dietary patterns and sociodemographic/lifestyle characteristics

Characteristics	Dietary patterns					
	Processed meat/ westernized	Fish/traditional	Fruit/vegetables	Reindeer/traditional	Bread and sandwich spreads	Sweets and bakery goods
	β (95% CI)	β (95% CI)	β (95% CI)	β (95% CI)	β (95% CI)	β (95% CI)
Age (years)						
40–49	Reference	Reference	Reference	Reference	Reference	Reference
50–59	−0.88*** (−0.99, −0.77)	0.56*** (0.44, 0.67)	0.26*** (0.14, 0.38)	−0.06 (− 0.16, 0.04)	0.03 (− 0.06, 0.12)	0.18*** (0.08, 0.29)
60–69	−1.34*** (−1.46, −1.22)	0.92*** (0.80, 1.05)	0.30*** (0.18, 0.43)	− 0.17** (− 0.28, − 0.07)	0.01 (− 0.09, 0.11)	0.45*** (0.34, 0.56)
P for linear trend	< 0.001	< 0.001	< 0.001	0.002	NS	< 0.001
Gender						
Men	Reference	Reference	Reference	Reference	Reference	Reference
Women	0.07 (−0.01, 0.16)	−0.47*** (− 0.56, − 0.38)	1.36*** (1.27, 1.45)	−0.09* (− 0.17, − 0.01)	−0.53*** (− 0.60, − 0.46)	0.14*** (0.06, 0.23)
Ethnic/geographic group						
Inland and coastal non-Sami	Reference	Reference	Reference	Reference	Reference	Reference
Inland Sami	−0.44*** (− 0.56, − 0.32)	−0.53*** (− 0.65, − 0.41)	−0.15* (− 0.28, − 0.03)	2.08*** (1.98, 2.19)	0.14** (0.05, 0.24)	0.07 (− 0.04, 0.18)
Inland multiethnic Sami	−0.30* (− 0.55, − 0.05)	−0.32* (− 0.58, − 0.06)	0.06 (− 0.21, 0.33)	1.42*** (1.19, 1.64)	0.15 (− 0.06, 0.36)	−0.13 (− 0.36, 0.10)
Coastal Sami	− 0.19* (− 0.33, − 0.04)	−0.03 (− 0.18, 0.12)	0.39*** (0.23, 0.54)	0.71*** (0.57, 0.84)	0.11 (− 0.01, 0.23)	−0.27*** (− 0.41, − 0.14)
Coastal multiethnic Sami	−0.02 (− 0.15, 0.11)	0.17* (0.04, 0.31)	0.05 (− 0.09, 0.18)	0.30*** (0.18, 0.42)	0.09 (− 0.02, 0.20)	−0.25*** (− 0.37, − 0.13)
Education level (years)						
0–9	Reference	Reference	Reference	Reference	Reference	Reference
10–12	0.33*** (0.21, 0.45)	0.02 (−0.10, 0.15)	0.14* (0.01, 0.27)	−0.29*** (− 0.40, − 0.18)	−0.10* (− 0.20, − 0.004)	−0.31*** (− 0.42, − 0.20)
13–16	0.35*** (0.23, 0.48)	0.09 (− 0.04, 0.22)	0.38*** (0.25, 0.52)	−0.25*** (− 0.36, − 0.13)	−0.29*** (− 0.39, − 0.18)	−0.41*** (− 0.53, − 0.30)
≥ 17	0.32*** (0.18, 0.47)	0.25*** (0.10, 0.40)	0.62*** (0.46, 0.77)	−0.19** (− 0.32, − 0.06)	−0.45*** (− 0.57, − 0.33)	−0.47*** (− 0.61, − 0.34)
P for linear trend	< 0.001	0.001	< 0.001	0.01	< 0.001	< 0.001
Gross annual household income (NOK)						
Low (≤450,000)	Reference	Reference	Reference	Reference	Reference	Reference
Average (451000–750,000)	0.16** (0.05, 0.27)	0.047 (−0.07, 0.16)	0.10 (−0.02, 0.22)	− 0.17** (− 0.27, − 0.07)	0.01 (− 0.08, 0.10)	−0.19*** (− 0.30, − 0.09)
High (> 750,000)	0.36*** (0.24, 0.49)	0.10 (− 0.03, 0.23)	0.19** (0.06, 0.32)	−0.35*** (− 0.46, − 0.24)	−0.14** (− 0.24, − 0.04)	−0.31*** (− 0.43, − 0.19)
P for linear trend	< 0.001	NS	0.005	< 0.001	0.009	< 0.001
Household size						
1	Reference	Reference	Reference	Reference	Reference	Reference
2	0.10 (−0.03, 0.23)	0.13* (0.0001, 0.26)	−0.07 (− 0.21, 0.06)	0.0008 (− 0.11, 0.12)	−0.04 (− 0.14, 0.07)	0.19** (0.07, 0.30)
3–4	0.24** (0.10, 0.38)	−0.17* (− 0.32, − 0.03)	−0.15 (− 0.30, 0.004)	0.16* (0.04, 0.29)	0.04 (− 0.08, 0.16)	0.32*** (0.18, 0.45)
5–8	0.17 (− 0.02, 0.36)	−0.38*** (− 0.57, − 0.18)	−0.17 (− 0.38, 0.03)	0.35*** (0.18, 0.52)	0.04 (− 0.12, 0.20)	0.54*** (0.36, 0.71)
P for linear trend	0.03	< 0.001	NS	< 0.001	NS	< 0.001
Physical activity level						
Low	Reference	Reference	Reference	Reference	Reference	Reference
Medium	−0.12* (−0.23, − 0.01)	0.07 (− 0.04, 0.18)	0.29*** (0.18, 0.40)	− 0.12* (− 0.21, − 0.02)	−0.06 (− 0.15, 0.03)	0.02 (− 0.08, 0.12)
High	−0.37*** (− 0.51, − 0.23)	0.10 (− 0.04, 0.25)	0.70*** (0.55, 0.85)	−0.07 (− 0.20, 0.05)	−0.16** (− 0.28, − 0.04)	−0.09 (− 0.22, 0.04)
P for linear trend	< 0.001	NS	< 0.001	NS	0.007	NS
Smoking status						
Never	Reference	Reference	Reference	Reference	Reference	Reference
Former	0.12** (0.03, 0.21)	0.24*** (0.15, 0.34)	0.01 (−0.09, 0.11)	−0.05 (− 0.13, 0.04)	−0.04 (− 0.12, 0.03)	−0.29*** (− 0.38, − 0.21)
Current	0.22*** (0.11, 0.34)	0.34*** (0.22, 0.46)	− 0.55*** (− 0.68, − 0.43)	0.23*** (0.13, 0.34)	0.23*** (0.13, 0.33)	−0.38*** (− 0.49, − 0.27)
BMI category						
Underweight/normal weight	Reference	Reference	Reference	Reference	Reference	Reference
Overweight	0.19*** (0.09, 0.29)	0.15** (0.05, 0.26)	0.06 (−0.05, 0.17)	−0.01 (− 0.10, 0.08)	−0.05 (− 0.14, 0.03)	−0.16** (− 0.25, − 0.06)
Obesity	0.20*** (0.09, 0.31)	0.16** (0.04, 0.28)	0.23*** (0.11, 0.35)	0.11* (0.004, 0.21)	0.04 (−0.05, 0.14)	−0.36*** (− 0.46, − 0.25)
P for linear trend	0.001	0.007	< 0.001	0.04	NS	< 0.001
R-sq	0.47	0.38	0.30	0.38	0.45	0.26

*CI* confidence interval. ^a^ Predictors were mutually adjusted each for other and for energy intake

* *p* < 0.05, ** *p* < 0.01, *** *p* < 0.001

NS-non significant, *p* ≥ 0.05

#### ‘Processed meat/westernized’

Inland Sami had the lowest scores for this pattern. A strong negative association was observed between age and ‘processed meat/westernized’ pattern scores. Older people (60–69 years) were less likely to report adherence to a ‘processed meat/westernized’ pattern. Those with the lowest education level (0–9 years), compared with the other three education groups, those who reported a high gross annual household income, participants with a low physical activity level, current smokers, and individuals with overweight/obesity were more likely to adhere to the ‘processed meat/westernized’ pattern.

#### ‘Fish/traditional’

Participants with high scores for the ‘fish/traditional’ pattern were more likely to be coastal multiethnic Sami, and were less likely to be inland Sami and inland multiethnic Sami. Being older, being a man, having ≥17 years of education, having a small household size, former and current smoking, and overweight/obesity were positively associated with the ‘fish/traditional’ pattern.

#### ‘Fruit/vegetables’

High education was a strong predictor of the ‘fruit/vegetables’ pattern. Participants aged 60–69 years, women, and those who reported a high physical activity level showed a greater adherence to this pattern. Smokers were less likely to report a diet rich in fruit and vegetables. The ‘fruit/vegetables’ pattern was positively associated with obesity.

#### ‘Reindeer/traditional’

The ‘reindeer/traditional’ pattern was strongly and positively associated with being inland Sami, followed by inland multiethnic Sami, coastal Sami, and coastal multiethnic Sami. Further, large household size, being a current smoker, and obesity were associated with increasing scores for the ‘reindeer/traditional’ pattern.

#### ‘Bread and sandwich spreads’

The ‘bread and sandwich spreads’ pattern was negatively associated with female gender, high education level, high gross annual household income, and high physical activity level. This pattern was positively associated with being a current smoker and being inland Sami.

#### ‘Sweets and bakery goods’

The ‘sweets and bakery goods’ pattern was positively associated with older age, female gender, and large household size. This pattern was negatively associated with high education level, high gross annual household income, current smoking, and obesity. Lower levels of adherence to this pattern were observed in coastal Sami and multiethnic Sami than in non-Sami; however, no ethnic differences were found between inland Sami/inland multiethnic Sami and non-Sami.

## Discussion

In this cross-sectional study of a large sample of Sami, multiethnic Sami, and non-Sami men and women in Northern Norway, we identified six independent dietary patterns with clear interpretability. The dietary patterns in the present study were related to sociodemographic and lifestyle characteristics of the study sample, including ethnic and geographic factors.

The two traditional dietary patterns – ‘fish/traditional’ and ‘reindeer/traditional’ – are unique to Northern Norway [2, 3]. The fruit/vegetables’ and the ‘processed meat/westernized’ patterns are also aligned with findings from previous studies and characterized by typical foods [36–38]. The ‘fruit/vegetables’ pattern was more reflective of healthy eating guidelines and appears to be the most health promoting pattern in the present study. The ‘processed meat/westernized’ and ‘sweets and bakery goods’ patterns contained a combination of foods that, in previous studies, have been associated with chronic diseases. In fact, sweets and bakery goods may also contribute to a so-called ‘western’ pattern, together with red meat/processed meat, and starchy and processed salty foods like snacks [39]. However, in the present study, high intake of sweets and bakery goods emerged as a distinct dietary pattern. The ‘bread and sandwich spreads’ pattern is a traditional breakfast and lunch pattern in Norway, comprising milk, coarse bread, fat on bread, cheese, and cured meats. A similar pattern has been previously identified in Norwegian women [40, 41].

Several reviews have demonstrated that consumption of healthier foods, such as whole grains, lean meat, fish, low-fat dairy products, and fresh vegetables and fruit, are more likely to be consumed by groups with higher SES. Conversely, consumption of less healthy foods, for instance, refined grains and added fats, have been associated with lower SES [42–44]. In the present study, older participants and participants with a higher education level, regardless of ethnicity, were more likely to adhere to what would be considered healthy dietary patterns, such as those with higher loadings of fish/seafood and fruit/vegetables, similar to the prudent/healthy pattern in previous studies [40, 45].

The ‘processed meat/westernized’ pattern explained the largest variance in food intake in the study sample, i.e., 7.3%. It has been reported that, in some high-income countries, people with lower SES may consume red and processed meat more often and in larger quantities [46]. However, we did not observe clear associations between lower SES and the ‘processed meat/westernized’ pattern. Interestingly, the other unhealthy dietary pattern in our study, ‘sweets and bakery goods’, was clearly associated with low education level, low gross annual household income, and large household size, which is in line with other international studies [17, 47, 48]. The ‘fruit/vegetables’ pattern was positively correlated with higher education level, gross annual household income, health-conscious lifestyle, and female gender, which is also in agreement with the previous Norwegian study [3]. In the present study, the ‘reindeer/traditional’ pattern correlated positively with less favorable economic conditions (low income and high household size); however, it was not positively correlated with older age like the ‘fish/traditional’ pattern. This result has not been previously shown, and additional research, including qualitative findings to confirm and explain these results, would be helpful.

The present study makes a valuable contribution to our understanding of multiethnic food culture in Northern Norway and the role of ethnicity and geography. When compared with our non-Sami reference group, the inland Sami group scored significantly higher on the ‘reindeer/traditional’ pattern and significantly lower on the ‘processed meat/westernized’ and ‘fish/traditional’ patterns. This result has been confirmed in analyses of ethnic differences in food [8] and nutrient intakes [7] in this population. In the present study, both multiethnic self-identification and coastal region of residence were related to lower levels of adherence to the ‘reindeer/traditional’ dietary pattern. No differences in adherence to the ‘processed meat/westernized’ pattern appeared between coastal multiethnic Sami and non-Sami, but lower levels of adherence to the ‘processed meat/westernized’ pattern were identified in inland Sami, followed by inland multiethnic Sami and coastal Sami. Some of the reasons for these differences could be: (i) historically, reindeer herding occurs primarily in the inland regions; (ii) the consequences of assimilation policies were most striking in the coastal regions, whereas Sami in the inland regions managed to preserve more of their culture. We also found that coastal multiethnic Sami, compared to non-Sami, showed somewhat larger adherence to the ‘fish/tradition’ pattern, which included lean fish and fish liver, while inland Sami and inland multiethnic Sami showed lower adherence to this pattern. The ‘fish/traditional’ pattern is unique to the coastal region of Northern Norway. To our knowledge, there is only one study that described a similar ‘fish’ pattern: a study conducted in adults residing in the isolated Canadian province of Newfoundland and Labrador, where cod fishery is also historically important [49].

Central obesity rates are high in both Sami and non-Sami in rural Northern Norway [21, 50]. BMI was related to dietary patterns in the present study, but the influence of BMI was weaker than that of other factors. We observed positive associations between obesity and healthy dietary habits (the ‘fruit/vegetables’ and the ‘fish/traditional’ patterns) and negative associations between obesity and unhealthy dietary habits (the ‘sweets and bakery goods’ pattern). Indeed, a similar weak positive association between higher BMI and the “prudent” dietary pattern was recently reported in a large cross-sectional study of dietary patterns in Norwegian women [36]. One explanation may be that people with a high BMI tend to underreport their intake of unhealthy foods, like sweets and bakery goods, and overreport their intake of healthy foods, like fruit/vegetables and fish [51]. We should also take into account the limitations of the cross-sectional design of the present study, for example, the antecedent-consequent bias i.e., we don’t know how exposure and outcome are time-related. Factors other than over- and underreporting may also have influenced the observed associations between BMI and dietary patterns, but given the complexity of this issue, more data is needed to explain these associations.

The literature states that adherence to some traditional diets, such as the Mediterranean diet, is associated with lower BMI [52]. In the present study, we observed positive associations between higher BMI and both the ‘processed meat/westernized’ and the ‘reindeer/traditional’ patterns, which has been previously reported in the population of rural Northern Norway [3]. One explanation for the latter finding might be that the traditional diet is currently highly mixed with processed food [8]. It was recently shown that ethnic differences in nutrient intake between Sami and non-Sami are small, that Sami women derived more energy from added sugars than non-Sami women, and that the inland population tended to have a higher intake of added sugars than the coastal population [7].

Another issue is that we used BMI to classify overweight and obesity. Although BMI is a widely used measure of adiposity in large epidemiological studies, there is an ongoing discussion in the literature about its appropriateness as a phenotypic marker of adiposity in populations with different ethnicities [53, 54]. For instance, Andersen et al. suggested the use of a higher BMI cut-off among the Inuit (the Arctic Indigenous population) than the non-Inuit population in Greenland [55]. Unfortunately, no specific guidelines with respect to BMI cut-off values exist for the Sami population. Consequently, using standard BMI cut-off values from the World Health Organization may overestimate the number of individuals with overweight and obesity in the Sami population.

Few studies have assessed the associations between dietary patterns and health outcomes in Arctic Indigenous people, and even fewer used a prospective design [56]. However, one study from Canada did both, and in that study, the Beef and Processed Food pattern, which was derived using factor analysis, was associated with an increased risk of incident type 2 diabetes in an Indigenous Canadian population, while the Balanced Marked Foods and Traditional Food patterns were not [56]. The results from the Canadian report provide evidence of the importance of dietary patterns in the development of chronic disease in Indigenous people; however, more studies are still required.

PCA and cluster analysis are two commonly used methods to derive dietary patterns. PCA uses the covariance matrix of the food groups to reduce the dimensionality from a high number of food groups to few patterns of food consumption [57]. Cluster analysis groups individuals with similar dietary patterns based on the mean of the food intake variables, and is able to identify key dietary patterns comparable with PCA [17]. Dietary patterns in the present study and in SAMINOR 1, which were conducted about a decade apart (2003–3004), were derived using different statistical methods [3]. Nevertheless, it appears that the major dietary patterns and their predictors are comparable with previous findings. Thus, the results of this study suggest that dietary patterns remained relatively stable in the rural population in Northern Norway during this time window. If so, this finding is in contrast to the rapid nutrition transition described in other Arctic Indigenous populations [58, 59]. The similarities in diet over time in Northern Norway could indicate that the rapid nutrition transition described in other Arctic Indigenous populations occurred earlier in the assimilation period among the Norwegian Sami. However, dietary patterns are not sensitive enough to detect small changes; only a major dietary pattern shift (i.e., major changes in the factor loadings) can be detected.

Some limitations of our study deserve to be mentioned. First, approximately half (47%) of the invited individuals participated in the survey. Response rates differed across age groups, genders, and municipalities, with a better response among older participants, women, and participants who live in the Kautokeino municipality (54%) [1]. Men younger than 50 years of age were relatively underrepresented. Also, only 10 municipalities were included in the present study. Therefore, the possibility of selection bias should be considered. Second, the sample was limited to the age group 40–69 years, thus our results cannot be applied to young adults or the elderly. Third, the pattern analysis may only capture limited portions of the overall diet: in the present study it was 28%. Nonetheless, other studies that used similar analyses in Norway and Denmark reported smaller or comparable proportions [15, 36, 41]. Fourth, PCA involves several subjective decisions, including the merging of food items into food groups prior to the analysis, the number of factors to retain, the method of rotation, the cut-off value used to define a significant contribution of the factor loadings, and self-interpretation/self-labelling of the factor components [60]. Fifth, in the present study, we relied on self-reported dietary intake, which requires participants to recall their dietary habits in the 12 months before the investigation. Hence, we cannot rule out recall, education, and social desirability biases. Moreover, we do not have information on whether or not there were ethnic differences in the degree of these biases. Another relevant limitation is that FFQ has not been specifically validated in men or in the Indigenous Sami population. However, the FFQ was validated for the general female population of Norway in several studies [32–34]. The use of a validated questionnaire developed for the majority population adapted to the Indigenous diet is very common in large epidemiological studies when a validation study is not feasible. We included the most frequently consumed local food items in our FFQ based on existing knowledge [2, 3, 61]; thus, the FFQ was adapted to suit the population being sampled. We previously reported than the total energy intake in Sami men living in inland areas was lower than that in non-Sami men [7]. It is possible that Sami men (especially in the inland region) were more likely to underreport their food intake in the present study or that the FFQ did not include some of the traditional food items they consumed. This may have resulted in a less accurate assessment of dietary intake in Sami participants and the loss of information about some additional components of traditional dietary patterns. In a previous study on the same sample, concentration of serum 25(OH) D and vitamin D intake were positively associated, proving to some extent the validity of dietary assessment [29]. Ethnic differences reflect cultural influences on dietary behaviors. However, food choice is complex and can be affected by many other social determinants, such as family, living alone, social support, and social setting, which may subsequently affect dietary patterns [62]. However, quantifying the social influence on food intake is difficult, and our study was not designed to answer this research question. A qualitative study that explores what kind of social factors are important and how Sami make decisions on food choice in practice would be helpful.

An important strength of the present study is the large number of study participants. Our rural population sample is unique and heterogeneous with respect to gender, ethnicity, geographic region of residence, education level, income, and lifestyle. The SAMINOR 2 Clinical Survey is a follow-up of SAMINOR 1, which included only a limited number of dietary questions. In contrast, the SAMINOR 2 Clinical Survey questionnaire was much more comprehensive and provides a better assessment of diet. In Norway, only a few large population-based studies have focused on dietary patterns, and the study samples included only women [36, 40, 41]. Unlike the previous studies, the SAMINOR sample includes both men and women, and we had the possibility to compare dietary patterns between two waves of the SAMINOR Study.

## Conclusions

The present study provides important insight into different dietary patterns and related sociodemographic and lifestyle factors in the multiethnic population of rural Northern Norway. Unhealthy lifestyle choices, like low physical activity level and smoking, and lower SES were associated with unhealthy dietary habits in the present study. Inland Sami were most strongly associated with the ‘reindeer/traditional’ pattern. We did not aim to observe the degree of stability of dietary patterns over time; instead we compared our results to those of previous studies on dietary patterns. Nevertheless, our results seem to generally support the hypothesis that dietary patterns in this population have remained relatively stable. This study is important for future analysis of dietary patterns and disease risk within the Sami population.

## Supplementary information

**Additional file 1: Table S1**. Description of the 53 food groups used in the analysis of dietary patterns.

## Data Availability

The datasets generated and/or analysed during the current study are not publicly available because the authors do not have permission to share data.

## Abbreviations

BMI: Body mass index
FFQ: Food frequency questionnaire
PCA: Principal component analysis
SES: Socioeconomic status.
the NOWAC study: the Norwegian Women and Cancer study
the SAMINOR Study: The Population-based Study on Health and Living Conditions in Regions with Sami and Norwegian Populations
