# Supplementary material for: Dietary patterns of adults and their associations with Sami ethnicity, sociodemographic factors, and lifestyle factors in a rural multiethnic population of northern Norway - the SAMINOR 2 clinical survey
Source: BMC Public Health. 2019 Dec 4;19:1632. doi: 10.1186/s12889-019-7776-z (PMC6894324; doi:10.1186/s12889-019-7776-z)
Supplement: Supplementary file 1 — Additional file 1: Table S1. Description of the 53 food groups used in the analysis of dietary patterns. [file 12889_2019_7776_MOESM1_ESM.docx]

Table S1

**Description of the 53 food groups used in the analysis of dietary patterns**

| **Food group** | **Item** |
| --- | --- |
| 1. Whole milk (regular or sour/fermented/cultured) | Drinking milk (regular or sour/fermented/cultured): whole milk (full fat, 4% fat) (1) |
| 2. Low-fat/skimmed milk (regular or sour/fermented/cultured) | Drinking milk (regular or sour/fermented/cultured): low fat milk (1.2%-1.8% fat) (1), extra low fat milk (1% fat) (2), skimmed milk (less than 0.3% fat) (3) |
| 3. White cheese, full and low fat | White cheese full fat (1), white cheese low fat (2) |
| 4. Whey cheese, full and low fat | Whey cheese full fat (1), whey cheese low fat (2) |
| 5. Milk or cream to coffee or tea | Milk or cream to coffee (1), milk or cream to tea (2) |
| 6. Full fat and low fat sour cream for fish meals/dishes | Sour cream 35% fat (1), sour cream 20% fat (2) |
| 7. Yogurt | Yogurt (1) |
| 8. Breakfast cereals | Breakfast cereal, oats/oatmeal or muesli (1) |
| 9. Rice porridge | Rice porridge (1) |
| 10. Other porridge; oatmeal, etc. | Other porridges, oatmeal, etc. (1) |
| 11. Coarse/semi coarse bread | Coarse bread (1), semi coarse bread (2) |
| 12. Crispbread | Crispbread, etc. (1) |
| 13. White bread | White bread (baguette) (1) |
| 14. Fat as spread on bread (butter, margarine and their blends) | Hard margarine (e.g. Melange) (1), soft margarine (e.g. Soft, Vita) (2), butter and margarine blends (e.g. Bremykt) (3), “Brelett” (fat reduced butter and margarine blend) (4), butter (5) |
| 15. Fat as spread on bread (light/olive oil margarine) | Reduced fat margarine (e.g. Soft light, Vita Lett) (1), olive oil margarine (e.g. Brelett oliven, Soft oliven) (2) |
| 16. Bacon, preserved meats (salami, ham, etc.), liver pâté (topping on bread) | Bacon/lard (1), sandwich meats, full fat content (salami, cured mutton, etc.) (2), sandwich meats, low fat (boiled ham, etc.) (3), liver pâté (4) |
| 17. Mayonnaise-based salads (sandwich spreads/fillings) | Mayonnaise-based salads, like prawn salad, Italian salad, etc. (1) |
| 18. Vegetables | Carrots (1), cabbage (2), swede (3), broccoli/cauliflower (4), mixed salad (5), tomato (6), frozen vegetable mix (7), onion (8), beans (9), peas (10), other vegetables (11) |
| 19. Potatoes | Boiled (1), mashed (2), pan-fried/fried (3) |
| 20. Fruit and berries | Apples/pears (1), oranges (2), bananas (3), other fruits (4), strawberries (fresh, frozen) (5), other berries (fresh, frozen) (6) |
| 21. Lean fish (filets, steaks) | Boiled cod/coalfish/haddock/pollack (1), pan-fried cod/coalfish/haddock/pollack (2), wolffish/flounder/redfish (3), halibut (4) |
| 22. Oily fish, filets/steaks and fish/spreads on bread (smoked/canned) | Salmon/sea trout (1), mackerel (2), herring (3); fish spreads: mackerel in tomato sauce/smoked mackerel (4), salmon (gravlax/smoked) (5), herring/anchovies (6) |
| 23. Freshwater fish | Freshwater fish (e.g. perch, grayling, pike, arctic charr, common whitefish, trout) (1) |
| 24. Fish products | Fishcakes/fish pudding/fish balls (1), fish stew/fish gratin (2), fried fish/fish sticks (3), other fish products/dishes (4), cod roe spread (“kaviar” in Norwegian) (5) |
| 25. Shellfish (i.e., prawns/shrimp, crabs, mollusks) | Shellfish (1) |
| 26. Fish roe/liver | Fish roe (1), fish liver (2) |
| 27. Liquid (bottled) cod liver oil | Liquid (bottled) cod liver oil (“tran” in Norwegian) (1) |
| 28. Seagull eggs or eggs of other seabirds | Seagull eggs or eggs of other seabirds (1) |
| 29. Red meat (beef, pork, and mutton) | Steak (beef, pork, mutton) (1), chops (beef, pork, mutton) (2), roast (beef, pork, mutton) (3) |
| 30. Reindeer meat | Reindeer meat (1) |
| 31. Game meat (moose meat, grouse, and other game birds) | Moose meat (1), grouse, other game birds (2) |
| 32. Food made with animal blood | Food made with animal blood, ie. black pudding (lamb/sheep, cattle, reindeer, moose) (1) |
| 33. Pizza with meat toppings | Pizza with meat toppings (1) |
| 34. Processed meat/meat dishes | Minced meat hamburger/meat patties (1), hot dogs (sausages) (2), meat casserole, stew (“lapskaus” in Norwegian) (3), other meat dishes (4) |
| 35. Sauces for fish, meat/pasta dishes | Sauce, high fat content (white/brown) for fish meals/dishes (1), sauce, fat free (white/brown) for fish meals/dishes (2), brown sauce for meat/pasta (3), gravy for meat/pasta (4), tomato-based sauce for meat/pasta (5), sauce containing cream/sour cream for meat/pasta (6) |
| 36. Melted/solid butter/margarine for fish meals/dishes | Melted/solid butter (1), melted/solid margarine (2) |
| 37. Chicken | Chicken with skin (1), chicken without skin (2) |
| 38. Pasta and rice | Pasta (spaghetti/macaroni/noodles) (1), rice (2) |
| 39. Soup | Soup as appetizer, lunch or supper (1), soup main dish (2) |
| 40. Eggs | Eggs (1) |
| 41. Bakery goods | Yeasted bakery goods (buns, etc.) (1), Danish pastries (2), cakes (3), pancakes (4), waffles (5), sweet biscuits (6), a traditional Norwegian soft bread/mashed potato flatbread (“lefser/lomper” in Norwegian) (7) |
| 42. Sweets, ice cream, chocolate, desserts | Chocolate/caramel pudding (1), rice pudding/creamed rice, mousse/fromage (2), compote/stewed fruit/canned fruit (3), jam, sandwich spread (4), milk chocolate (5), dark chocolate (6), ice cream in the summer (9), ice cream, the rest of the year (10), sweets/candy (“godteri“ in Norwegian) (11) |
| 43. Sugar to coffee and tea | Sugar (not including artificial sweeteners) to coffee (1), sugar to tea (2) |
| 44. Salty snacks | Potato chips (1), peanuts (2), other nuts (3), other snacks (4) |
| 45. Water (tap/bottled) | Tap water (1), bottled water (2) |
| 46. Coffee, except boiled | Filtered coffee (1), espresso (2), latte (3), instant coffee (4) |
| 47. Unfiltered/boiled coffee | Unfiltered/boiled coffee or plunger/steeped coffee (1) |
| 48. Juice | Orange juice (1), other fruit juices (2) |
| 49.Squash/lemonade/soft drink containing sugar and without sugar | Squash/lemonade/soft drink containing sugar (1), squash/lemonade/soft drink without sugar (2) |
| 50. Beer/alcopops | Beer/alcopops (500 ml) (1) |
| 51. Wine | Wine (glass) (1) |
| 52. Liqueur/fortified wine | Liqueur/fortified wine (glass) (1) |
| 53. Liquor/distilled spirits | Liquor/distilled spirits (drink, shot) (1) |
